# Effectiveness of Unihemispheric Concurrent Dual-Site Stimulation over M1 and Dorsolateral Prefrontal Cortex Stimulation on Pain Processing: A Triple Blind Cross-Over Control Trial

**DOI:** 10.3390/brainsci11020188

**Published:** 2021-02-04

**Authors:** Francisco Gurdiel-Álvarez, Yeray González-Zamorano, Sergio Lerma Lara, Julio Gómez-Soriano, Julian Taylor, Juan Pablo Romero, María Gómez Jiménez, Josué Fernández-Carnero

**Affiliations:** 1 Escuela Internacional de Doctorado, Department of Physical Therapy, Occupational Therapy, Rehabilitation and Physical Medicine, Universidad Rey Juan Carlos, 28933 Alcorcón, Spain; franfisiotmno@gmail.com (F.G.-Á.); yeraygonzalezzamorano@gmail.com (Y.G.-Z.); 2 Department of Physical Therapy, Centro Superior de Estudios Universitarios La Salle, Universidad Autónoma de Madrid, 28023 Madrid, Spain; sergio.lerma@lasallecampus.es (S.L.L.); maria.gomez.jimenez@lasallecampus.es (M.G.J.); 3 Motion in Brains Research Group, Institute of Neuroscience and Sciences of the Movement (INCIMOV), Centro Superior de Estudios Universitarios La Salle, Universidad Autónoma de Madrid, 28023 Madrid, Spain; 4 Toledo Physiotherapy Research Group (GIFTO), Faculty of Physiotherapy and Nursing, Universidad Castilla La Mancha, 45071 Toledo, Spain; Julio.Soriano@uclm.es; 5 Sensorimotor Function Group, Hospital Nacional de Parapléjicos, SESCAM, 45071 Toledo, Spain; juliantaylorgreen2@gmail.com; 6 Harris Manchester College, University of Oxford, Oxford OX1 3TD, UK; 7 Facultad de Ciencias Experimentales, Universidad Francisco de Vitoria, 28223 Pozuelo de Alarcón, Spain; p.romero.prof@ufv.es; 8 Brain Damage Unit, Beata María Ana Hospital, 28007 Madrid, Spain; 9 Department of Physical and Occupational Therapy, Rehabilitation and Physical Medicine, Universidad Rey Juan Carlos, 28922 Madrid, Spain; 10 La Paz Hospital Institute for Health Research, IdiPAZ, 28046 Madrid, Spain; 11 Grupo Multidisciplinar de Investigación y Tratamiento del Dolor, Grupo de Excelencia Investigadora, Universidad Rey Juan Carlos-Banco de Santander, 28922 Madrid, Spain

**Keywords:** conditioned pain modulation, temporal summation, transcranial direct current stimulation, primary motor cortex M1, dorsolateral prefrontal cortex, pain, healthy subjects

## Abstract

Background: Transcranial direct current stimulation (tDCS) of the motor cortex (M1) produces short-term inhibition of pain. Unihemispheric concurrent dual-site tDCS (UHCDS-tDCS) over the M1 and dorsolateral prefrontal cortex (DLPFC) has greater effects on cortical excitability than when applied alone, although its effect on pain is unknown. The aim of this study was to test if anodal UHCDS-tDCS over the M1 and DLPFC in healthy participants could potentiate conditioned pain modulation (CPM) and diminish pain temporal summation (TS). Methods: Thirty participants were randomized to receive a sequence of UHCDS-tDCS, M1-tDCS and sham-tDCS. A 20 min 0.1 mA/cm^2^ anodal or sham-tDCS intervention was applied to each participant during three test sessions, according to a triple-blind cross-over trial design. For the assessment of pain processing before and after tDCS intervention, the following tests were performed: tourniquet conditioned pain modulation (CPM), pressure pain temporal summation (TS), pressure pain thresholds (PPTs), pressure pain tolerance, mechanosensitivity and cold hyperalgesia. Motor function before and after tDCS intervention was assessed with a dynamometer to measure maximal isometric grip strength. Results: No statistically significant differences were found between groups for CPM, pressure pain TS, PPT, pressure pain tolerance, neural mechanosensitivity, cold hyperalgesia or grip strength (*p* > 0.05). Conclusions: Neither UHCDS-tDCS nor M1-tDCS facilitated CPM or inhibited TS in healthy subjects following one intervention session.

## 1. Introduction

Transcranial direct current stimulation (tDCS) has been defined as a non-invasive form of brain stimulation in which a relatively weak direct current is passed into the cerebral cortex through small scalp electrodes, obtaining a modulation of cortical excitability apparently dependent upon the polarity of the stimulation [1,2]. Therefore, tDCS is considered different from other brain stimulation techniques as it does not evoke action potentials [1]. The increase or decrease in cortical excitability depends on whether the active electrode is the anode or the cathode, respectively [2,3]. In healthy subjects, effects have been demonstrated on motor-evoked potentials [2,4,5], cerebral blood oxygen levels measured with functional magnetic resonance imaging [6] and regional cerebral blood flow [7,8]. In addition, other effects include increased conditioned pain modulation (CPM) [9], reduced temporal summation (TS) [10], modulation of mechanical pain threshold [9,11], warm and cold perception thresholds [12], hot and cold pain thresholds [10,12,13], acute thermal pain [14] and pain perception to electrical stimulation [15].

The electrode location is also a relevant parameter to consider. Many studies have applied tDCS over the primary motor cortex (M1) [3,4,5,6,7,8,9,10,11,12,13,15,16,17], which modulates several different types of cortical pain processing [9,15]. Other tDCS studies analyzed the effect of the dorsolateral prefrontal cortex (DLPFC) [15,18,19] because of its role in the cognitive and emotional aspects of pain [15]. Nevertheless, stimulation of M1 has been shown to be the best region to modulate brain excitability [20].

The DLPFC has a central role in pain processing [21]. Stimulation of the DLPFC may play a role in the regulation of pain tolerance [22] and also in an increase in the mechanical sensitivity threshold and the pain pressure threshold (PPT) [20]. When compared to M1 alone, simultaneous stimulation of the DLPFC induced a 50% larger modulation of M1 corticospinal excitability, which lasted up to 24 h [23]. The concept of unihemispheric concurrent dual-site tDCS (UHCDS-tDCS) has also been shown to be effective in the enhancement of motor learning and motor function [24,25]. However, the effect of UHCDS-tDCS [25] on the modulation of pain has not been studied.

The main objective of the present study was to evaluate the effect of UHCDS-tDCS of M1 and DLPFC, compared to M1 stimulation alone, on the endogenous pain inhibitory system [9], and on TS in response to repeated noxious stimuli [26]. The secondary objective was to evaluate the effect of UHCDS-tDCS on pressure pain threshold and tolerance, cold pain, a neural mechanosensitivity test and isometric grip strength.

## 2. Materials and Methods

The SPIRIT 2013 Checklist has been used to assure the quality of the study protocol [27].

### 2.1. Study Design and Participants

Ethical approval was given by the Universidad Rey Juan Carlos Ethics Committee (#30012020204820) following the Helsinki Declaration (revised version 2013). The study was registered at clinicaltrials.gov (NCT04432363). Participants were recruited from the Universidad Rey Juan Carlos (Madrid, Spain). All participants read the patient information sheet regarding the study and signed the informed consent form. Participants did not receive any financial compensation for participation in the study, and their contribution was free and voluntary.

- Inclusion criteria and recruitment

The inclusion criteria for participants were defined as: age between 18 and 40 years and comprehension of the study tasks. The exclusion criteria were defined as: presence of pain in the previous 6 months, altered sensitivity in the tested regions, intolerance to electrotherapy, presence of pacemakers or any other implanted electrical device, ulcers or scars on the skin at the location of the electrodes, treatment with direct current stimulation within one week of the study intervention, pregnancy, frequent headaches, epilepsy, history of neuromuscular disease, previous clinical history of cervical surgery, injuries or surgery affecting the upper limbs, diabetes mellitus, cardiovascular diseases, oncological diseases and consumption of analgesic drugs seven days before the study.

### 2.2. Intervention Protocols

The study was carried out in a laboratory at the University Clinic of the Universidad Rey Juan Carlos in Madrid, where ambient temperature was controlled within the range of 22–26 °C.

The experimental design consisted of a triple-blinded, sham-controlled, cross-over clinical trial. Participants were initially randomized into three groups that would receive either unilateral application tDCS at M1 (A), combined tDCS at M1 and DLPFC (UHCDS-tDCS (B) or sham-tDCS (C) with the same electrode configuration within the helmet to ensure the blinding, with a statistically powered target group size of *n* = 30. Following allocation to the M1, M1 + DLPFC or sham group, each subject received the next group intervention separated by at least 72 h following an order of ABC, BCA or CAB. The randomization was performed using GraphPad QuickCalcs at https://www.graphpad.com/quickcalcs/ by the researcher (F.G.A.) who was responsible for administration of the intervention.

The assessor (Y.G.Z.) performed all the sensory evaluation tests and was blinded to the tDCS condition (M1 stimulation, M1 + DLPFC stimulation or sham stimulation). The participants were also blinded to the tDCS condition (both for sham and for real conditions), which was ensured by the operator performing the 20 min tDCS intervention using the same equipment, identical electrodes with their cables and the same electrode location. The operator (F.G.A.) was blinded by the assessor (Y.G.Z.) using the Neuroelectrics^®^ Instrument Controller software blinding option (Neuroelectrics Barcelona, SLU, Spain), which allowed us to hide the stimulation protocol parameters with a password and give a code to each protocol (A, B or C). The operator (F.G.A.) was also blinded to the protocol which corresponded to each subject. To make sure that blinding was maintained in subjects, they were asked after each session if they thought they had received either a sham or active intervention.

All subjects participated in three 90 min sessions performed on different days. During the first session, the participants completed several questionnaires related to quality of life and psychological factors related to pain (anxiety, depression, pain catastrophizing, kinesiophobia, fear of pain, sleep quality and physical activity) [28]. Quantitative sensory testing including pain pressure thresholds, pain pressure tolerance, conditioned pain modulation, neural mechanosensitivity, cold hyperalgesia and grip strength testing were performed before each treatment session. Following the initial evaluation, the assigned 20 min tDCS intervention was applied, followed immediately by the test evaluation again.

- Direct current stimulation intervention

For both the sham and active stimulation sessions, the participants were placed in a relaxed sitting position. All the interventions lasted 20 min and a direct current stimulator (Starstim tCS^®^, Neuroelectrics, Barcelona, Spain) with one 25 cm^2^ sponge cathode and two 10 cm^2^ sponge anodes (MRI Sponstim, Neuroelectrics, Barcelona, Spain) were used, following application of a 0.9% saline solution (Serrasol Laboratorios, Serra Pamies, Reus, Spain). In all cases, the stimulation intensity was increased gradually over a period of 10 s until reaching the programmed intensity.

For M1 stimulation, a 10 cm^2^ anode placed at the C3 electrode position (10/20 EEG system), a reference 10 cm^2^ electrode placed at the F3 electrode position (10/20 EEG system) and the 25 cm^2^ cathode placed on the contralateral supraorbital zone (Fp2 on the 10/20 EEG system) were configured and 1 mA stimulation was applied. This configuration of electrodes has been shown to be effective for the stimulation of the primary motor cortex [29]. For UHCDS-tDCS, two anodes of 10 cm^2^ were placed at C3 and F3, while a 25 cm^2^ cathode was placed on Fp2. Stimulation was applied at 2 mA intensity. This novel UHCDS-tDCS configuration has been previously applied in studies in healthy subjects [11,23] and patients with stroke [25], stimulated with a current density of 0.1 mA/cm^2^ over the anode.

A sham 20 min tDCS intervention was carried out with the same electrode location using the same equipment. Participants received a combined M1 + DLPC application with 2 mA stimulation during the first 30 s, and then the stimulation was decreased to zero until the last 30 s of the 20 min session when the stimulation was increased again to 2 mA (this was controlled automatically by the stimulator). The active stimulation is short enough not to generate any effect and is considerably shorter than a 3 min tDCS, which has been shown not to influence cortical excitability [2,17,30], and which is often used as a sham-tDCS condition [31,32].

### 2.3. Outcomes Measurement

Sensory and strength measures were recorded before and after each tDCS intervention. All participants were instructed that they could stop the testing session when they wanted, especially if the controlled test pain stimuli could not be tolerated.

- Pressure pain threshold

To evaluate mechanical pain, a digital pressure algometer (Model FDIX, Wagner Instrument Mark), with a 1 cm diameter flat rubber probe, was applied perpendicular to the skin. Pressure was applied manually at approximately 0.5 kg/s [33], and the threshold force at which pain was perceived was recorded. The pressure pain threshold (PPT) was assessed at a point marked on the nail bed of the right thumb, and these locations have already been used in previous studies [34]. In addition, the PPT was assessed at the right upper trapezius, midway between the C7 spinous process and the acromion, and at the right anterior tibialis muscle, 5 cm distal to the anterior tibial tuberosity and 1 cm lateral to the tibial anterior border [35]. Three PPT measurements were recorded from each test site with a minimum interval of 10 s [36], so that the average threshold value could be calculated in kg/cm^2^. The reproducibility and reliability of the results obtained by algometry to measure the pressure pain threshold have been demonstrated previously [37].

- Widespread mechanical hyperalgesia

Widespread mechanical hyperalgesia was calculated by adding up the means of the three PPT locations [38]. Lower values of widespread mechanical hyperalgesia were associated with a more pronounced widespread mechanical hyperalgesia, while higher values indicated absence of widespread mechanical hyperalgesia.

- Temporal summation

Temporal summation (TS) was elicited with 10 applications of the algometer (Model FDIX, Wagner Instrument Mark) at the individual pressure pain threshold intensity perceived at the nail bed of the right thumb. TS was elicited 2 min following quantification of the PPT. This was performed to ensure that the TS was not affected by possible sensitization following estimation of the PPT. For each pulse, the pressure was increased at a rate of approximately 2 kg/s to the previously determined PPT intensity, where it was maintained for 1 s before being released [39]. Pulses were presented with an interstimulus interval of 1 s because this has previously been shown to be optimal for inducing TS with pressure pain [40]. Before application of the first pressure pulse, subjects were instructed to manually rate the pain intensity of the first and 10th pulse with a 10 cm visual analog scale (VAS). The VAS ranged from 0 to 10, with the 0-anchor labeled with “no pain” and the 10-anchor labeled as “worst imaginable pain”.

- Conditioned pain modulation

To evaluate conditioned pain modulation (CPM), a handheld pressure algometer was used as the test stimulus to evaluate the PPT before and after application of a conditioning stimulus. Ischemic muscle pain was used as the conditioning stimulus using a sphygmomanometer (Model Minimus^®^ II, Rudolf Riester, Jungingen, Germany). The sphygmomanometer was applied around the left upper arm, with its lower rim 3 cm proximal to the cubital fossa. The cuff was inflated to 260 mmHg and maintained until the subject perceived a 6/10 pain on the VAS scale [41]. Then the PPT was measured at the nail bed of the right thumb and the cuff pressure was released. After 1 min, another PPT measurement was taken to evaluate the sustained effect of the CPM [42].

#### Secondary Outcomes

- Pain pressure tolerance

To evaluate pain pressure tolerance, a digital pressure algometer (Model FDIX, Wagner Instrument Mark), with a 1 cm diameter flat rubber probe, was applied perpendicular to the skin. Pressure was applied manually at approximately 0.5 kg/s [33]. The test was completed once per subject and was stopped when participants were unable to tolerate the pressure or a cut-off value of 13.5 kg was reached. The pressure at which the subject was unable to tolerate more pressure was recorded. Pressure pain tolerance was assessed at a point marked on the nail bed of the right thumb.

- Neural tension test

The upper limb neural test (ULNT1) for the median nerve was performed on the right upper limb according to the operational definition described by Butler [43]. The patient was positioned supine without a pillow. The left hand rested on the participant’s abdomen. The assessor followed the sequence of shoulder girdle fixation, shoulder abduction, wrist extension, forearm supination, shoulder external rotation and elbow extension. Movements were performed to the end of range or until symptoms were produced [44,45]. Prior to performing the test, patients were instructed to communicate the onset of any sensation such as stretch, tingling or pain anywhere in the arm or neck. The elbow extension angle at which the symptoms appeared was measured with a goniometer.

- Cold pain intensity

Cold pain intensity was evaluated with the subject resting on a chair with the ice application test [46]. Testing was conducted on the anterior skin of the right forearm. For the ice application test, the ice pack was held on the skin for 10 s. After 10 s of ice application, subjects were instructed to rate the intensity of pain on a 10 cm VAS. The VAS ranged from 0 to 10, with the 0-end point labeled with “no pain” and the 10-end point labeled as “worst imaginable pain”. The procedure was repeated three times to obtain a mean value, with a 60 s rest period between measures to avoid temporal summation of pain [47].

- Maximal isometric grip strength

Hand grip strength was measured with an isometric grip test using a handheld analogic dynamometer (Saehan Hydraulic Hand Dynamometer, 0–90 kg). Subjects were positioned in a straight-backed chair with both feet on the floor and the forearm resting on the table. Each subject was instructed to assume a position of adducted and neutrally rotated shoulders. For the arm to be tested, the elbow was flexed to 90°, the forearm and wrist were in neutral positions, and the fingers were flexed as needed for a maximal contraction [48]. Subjects performed a maximal isometric grip contraction until they produced a maximal force output. Three measures were taken with a 1 min rest period between tests, and the mean value was recorded. Some previous studies have found that there is an improvement in manual pressure force after tDCS, and it is appropriate to measure it to assess whether it has not only sensory but also motor effects [49]. The mechanisms proposed for strength improvement after tDCS are an increment of corticospinal excitability, diminished short-interval intra-cortical inhibition and increased cross-activation [50,51,52]

- State–trait anxiety inventory

The Spanish version of the Spielberg State–Trait Anxiety Inventory (STAI) [53] is a 40-item self-report measure for anxiety using a 4-point Likert-type scale (from 0 to 3 points) for each item. It has two scales, one for state anxiety (STAI-S) and one for trait anxiety (STAI-T). Both scales consist of 20 items, with several reverse-scored items (10 in the STAI-S and 7 in the STAI-T).

- Beck Depression Inventory-II

The Spanish version of the 21-item self-administered Beck Depression Inventory-II (BDI-II) [54] assesses the severity of subjective depressive symptoms. Each response is scored on a scale from 0 (not) to 3 (severe). The test covers cognitive and somatic/vegetative symptoms.

- Pain Catastrophizing Scale

The Spanish version of the Pain Catastrophizing Scale (PCS) [55] assesses catastrophizing cognitions and behaviors in relation to pain in clinical and nonclinical populations. It has 13 items and each one is rated on a 5-point scale: 0 (not at all) to 4 (all the time).

- Tampa Scale for Kinesiophobia

The Spanish version of the Tampa Scale for Kinesiophobia (TSK) [56] has 11 items and assesses fear of injury or re-injury in relation to movement and physical activity. It is a 4-point Likert-type scale (from 1 to 4 points).

- Fear of Pain Questionnaire III

The Spanish version of the 30-item Fear of Pain Questionnaire (FPQ-III) [57] assesses fear of experiencing pain. In this questionnaire, respondents are asked to rate how fearful they are of experiencing each response item on a 5-point Likert-type scale ranging from 1 (not at all) to 5 (extreme).

- The Pittsburgh Sleep Quality Index

The Spanish version of the the Pittsburgh Sleep Quality Index (PSQI) [58] consists of 19 self-rated questions and five questions to be answered by bedmates or roommates. The self-rated items of the PSQI generate seven component scores (with subscales ranging from 0–3): sleep quality, sleep latency, sleep duration, habitual sleep efficiency, sleep disturbance, use of sleeping medication and daytime disfunction. The sum of these seven component scores yields one global score of subjective sleep quality (range 0–21). Higher scores represent poorer subjective sleep quality.

- International Physical Activity Questionnaire Short Form

The Spanish version of the International Physical Activity Questionnaire Short Form (IPAQ-SF) [59] uses 9 items to record the weekly activity of four intensity levels: (1) vigorous-intensity activity such as aerobics, (2) moderate-intensity activity such as leisure cycling, (3) walking and (4) sitting.

### 2.4. Sample Size Calculation

The minimal group size for the trial was calculated using G*Power software (University of Dusseldorf, Germany), taking into consideration the design of three intervention groups and two measurement times. The primary outcome was chosen to be CPM with an estimated effect size of F = 0.423 using the Flood et al. [9] trial as a reference. An α level of 0.05 and a power of 95% were assumed, with a total sample of 69 subjects estimated. Considering a 20% follow-up loss, we estimated a minimal sample size of 28 subjects for each of the intervention groups.

### 2.5. Data Analyses

SPSS version 25.0 for Windows (IBM Corp. Released 2013. IBM SPSS Statistics for Windows, Version 22.0. Armonk, NY: IBM Corp) was used for statistical analysis. A Kolmogorov–Smirnov test was used to confirm the normal distribution of data. A descriptive analysis was selected to summarize the outcomes in the 2 measurements from all groups (M1-tDCS group, UHCDS-tDCS group or sham-tDCS group). Data were expressed as mean ± SD. A one-way ANOVA with the factor group (M1-tDCS group, UHCDS-tDCS group or sham-tDCS group) was performed. We carried out a comparison of the psychological variables at the baseline to evaluate if there were differences between the subjects, but none were found.

Repeated measures analysis of variance (ANOVA) was performed considering the significance of the Greenhouse–Geisser correction when the Mauchly test rejected the sphericity. To compare the differences between the 3 groups at the baseline versus after treatment, a repeated measures ANOVA with 2 factors, 3 (groups) × 2 times (pre and post), was used. To include the calculation of the effect size, the partial eta (η2) coefficient was used.

All analyses were carried out following the “per intent-to-treat” approach. In addition, a *p*-value lower than 0.05 was considered as statistically significant for a 95% confidence interval (CI).

## 3. Results

### Demographic and Clinical Characteristics

**Figure 1:**
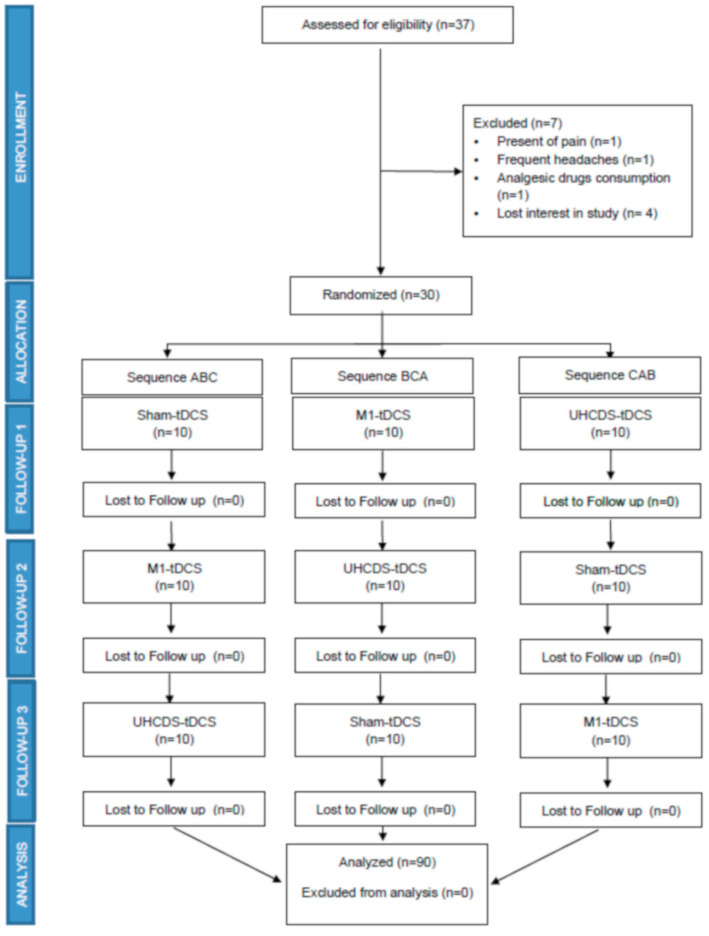
Consort flow diagram. tDCS: Transcranial direct current stimulation; UHCDS-tDCS: unihemispheric concurrent dual-site-Transcranial direct current stimulation.

Thirty-seven potential healthy subjects were initially recruited, of which 30 subjects met the inclusion criteria for recruitment into the clinical trial (see Figure 1 for the CONSORT flow diagram). A cross-over controlled trial was designed, and participants for the first session were randomized into one of three experimental groups: unilateral application tDCS at M1 (M1-tDCS, *n* = 10), combined tDCS at M1 and DLPFC (UHCDS-tDCS, *n* = 10) or sham-tDCS (*n* = 10). Three participants were excluded from the study because of either the presence of pain before the intervention, the presence of frequent headaches or treatment with analgesic drugs (see Figure 1). The mean age of the recruited subjects was 21.9 ± 2.8 years, height 1.70 ± 0.08, weight 66.2 ±10.3 Kg and BMI 22.68 ± 1.90, and the group consisted of 17 males (56.7%) and 13 females (43.3%, Table 1). Furthermore, no significant differences were identified for the main outcomes at the pre-intervention assessments between the three experimental intervention groups (Table 2). All participants tolerated the interventions well and the most frequent adverse effects were headache (12.1%), numbness (10%) and tickling sensations (8.1%). No severe adverse effects were reported. All participants were asked after each intervention if they believed they had received an active stimulation or a sham stimulus, so that blinding could be controlled effectively after the study. Only 53.3% of the subjects in the sham group were able to guess that they received the sham stimulation.

- Differences in the outcomes by gender

After performing the statistical analysis to evaluate the differences between males and females, no differences between the sexes were found for any of the variables, except for manual grip strength, where men showed a higher post-treatment strength (*p* = 0.023) and a greater improvement in cold hyperalgesia (*p* = 0.018), but only in the M1 group.

- Pressure pain threshold
- Pressure pain threshold (over finger)

The ANOVA showed neither a significant effect for time (F = 0.299; *p* = 0.586; η_p_^2^ = 0.003) nor a statistically significant interaction between group and time (F = 0.043; *p* = 0.958; η_p_^2^ = 0.001) for changes in the PPT measured in the finger (Table 2). The post hoc analysis did not reveal a significant difference between pre- and post-intervention (*p* > *0*.05 Table 2).

- Pressure pain threshold (trapezius muscle)

The ANOVA showed a significant effect for time (F = 3.924; *p* < 0.05; η_p_^2^ = 0.043) but not a statistically significant interaction between group and time (F = 0.006; *p* = 0.994; η_p_^2^ = 0.000) for changes in the PPT in the trapezius muscle (Table 2). The post hoc analysis did not reveal a significant within-group pre- and post-intervention difference (*p* > 0.05 Table 2).

- Pressure pain threshold over tibial muscle

The ANOVA showed a significant effect for time (F = 16.477; *p* < 0.0001; η_p_^2^ = 0.159) but no significant interaction between group and time (F = 1.397; *p* = 0.253; η_p_^2^ = 0.031) for changes in the PPT of the tibial muscle (Table 2). The post hoc analysis revealed significant within-group pre- and post-intervention differences only for the sham-tDCS and M1-tDCS groups, with a small effect size for the sham group (*p* = 0.035, d = −0.18) and M1-tDCS group (*p* = 0.001, d = −0.34, Table 2).

- Widespread mechanical hyperalgesia

The ANOVA showed a significant effect for time (F = 8.506; *p* < 0.01; η_p_^2^ = 0.089) but no statistically significant interaction between group and time (F = 0.269; *p* = 0.764; η_p_^2^ = 0.006) for the PPT summation (data presented in Table 2). The post hoc analysis revealed a significant within-group pre- and post-intervention difference only for the M1 group, with a small effect size (*p* = 0.030, d = −0.21) (Table 2).

- Temporal summation

For temporal summation, the ANOVA test neither showed a significant effect for time (F = 0.004; *p* = 0.951; η_p_^2^ = 0.000) nor a statistically significant interaction between group and time (F = 1.055; *p* = 0.353; η_p_^2^ = 0.024) (Table 2).

- Conditioned pain modulation

The ANOVA neither showed a significant effect for time (F = 3.551; *p* = 0.0631; η_p_^2^ = 0.039) nor a statistically significant interaction between group and time (F = 1.730; *p* = 0.183; η_p_^2^ = 0.038) for changes in conditioned pain modulation (Table 2). The post hoc analysis revealed a significant within-group pre- and post-intervention difference, but only for the sham-tDCS group with a low–medium effect size (*p* = 0.019, d = −0.45. (Table 2).

- Pain tolerance

The ANOVA test neither showed a significant effect for time (F = 0.302; *p* = 0.584; η_p_^2^ = 0.003) nor a statistically significant interaction between group and time (F = 0.965; *p* = 0.385; η_p_^2^ = 0.022) for pain tolerance (data presented in Table 2).

- Neural tension test

The ANOVA revealed significant differences for time (F = 19.431, *p* < 0.0001, η_p_^2^ = 0.183) but not for the group and time interaction (F = 0.610, *p* = 0.545, η_p_^2^ = 0.014) for the neural tension test (data presented in Table 2). The post hoc analysis revealed a significant within-group pre- and post-intervention difference, but only for the sham-tDCS and M1-tDCS groups, with a low effect size for the sham-tDCS group (*p* = 0.025, d = −0.21) and M1-tDCS group (*p* = 0.001, d = −0.30) (Table 2).

- Cold Hyperalgesia

The ANOVA showed a significant effect for time (F = 11.497; *p* < 0.001; η_p_^2^ = 0.119) but not a statistically significant interaction between group and time (F = 1.782; *p* = 0.175; η_p_^2^ = 0.040) for changes in cold hyperalgesia (data presented in Table 2). The post hoc analysis revealed significant within-group pre- and post-intervention differences, but only for the sham-tDCS and the M1-tDCS groups, with a low effect size for the sham-tDCS group (*p* = 0.029, d = −0.18) and the M1-tDCS group (*p* = 0.002, d = −0.30) (Table 2).

- Maximal isometric grip strength

The ANOVA showed a significant difference for time in the hand grip test (F = 4.635, *p* = 0.034, η_p_^2^ = 0.052) but not for the group*time interaction (F = 0.825, *p* = 0.442, η_p_^2^ = 0.019) for hand grip strength measurements. The post hoc analysis did not reveal a significant within-group pre- or post-intervention effect (*p* > 0.05) (Table 2).

## 4. Discussion

The aim of the current study was to assess the effect of UHCDS-tDCS at M1 and DLPFC on CPM and TS in healthy subjects. UHCDS-tDCS targeted over M1 and DLPFC was not effective in modulating CPM or TS in healthy subjects when compared to sham-tDCS. Additionally, conventional M1-tDCS failed to modulate CPM and TS in healthy subjects. These results contrast with the findings of previous studies [9,10,11,12,16,26,60].

An increase in inhibitory CPM in healthy subjects has been reported after high-definition tDCS (HD-tDCS) application [9,16]. HD-tDCS has a more focal effect on cortical excitability in the area directly under the central anode, and the brain modulation effect is restricted to within the area of stimulation [3]. In contrast, the conventional tDCS electrode montage can produce a more diffuse change in cortical excitability, focusing the induced electric fields between the stimulating electrodes [12,16]. In our study, we used two types of conventional tDCS montages, which could produce diffuse changes in cortical excitability in remote regions associated with M1, contributing to pain reduction [61]. Another difference between our study and a previous study was that the healthy cohort was composed of males only, which reduced the variability of the CPM effect and pain thresholds associated with gender [62]. Another difference from other studies is that the post-intervention test for assessing the effectiveness of blinding was not performed.

Conventional M1-tDCS has been shown to increase the CPM response in healthy subjects [10,11]. Although Reidler et al. [11] found an improvement in CPM, they used a parallel CPM protocol. The parallel protocol, in which the painful test stimulus and conditioned stimulus are presented simultaneously, has been argued to result in an exaggerated inhibitory response because of the distracting influence of the concurrent conditioning stimulus [63]. On the other hand, Braulio et al. [10] did not include females in the cohort and used a parallel model, which does not take into account a possible gender effect of CPM and its response to tDCS. In our study, we used a cross-over model to compare each subject with themselves between experimental groups, which reduced the variability of the CPM response. Although less efficient CPM has been reported in female subjects [62], we consider that a mixed sample is more representative of the clinical population; CPM deficiency in female subjects has been suggested to explain why greater chronic pain prevalence has been reported by this gender [62,64]. Caution should be taken when extrapolating changes in experimental pain observed in healthy subjects to clinical pain.

The use of handheld algometry or cuff algometry as the test stimulus, and the cold pressor task as the conditioning stimulus, has been proven to provide the most reliable CPM effect [65]. We used the cuff test as an ischemic conditioning stimulus, which could contribute to the difference in the results reported in other studies which used the cold pressor task as the conditioning stimulus [9,10,11]. Although other studies used a combination of cuff algometry as the conditioning stimulus and handheld algometry as the test stimulus [16], these studies also applied HD-tDCS instead of conventional tDCS. One randomized controlled double-blinded trial applied conventional tDCS, which used the cold pressor task as the conditioning stimulus and heat pain threshold as the test stimulus, but no increase in CPM was observed [4]. Another randomized controlled double-blinded trials applied similar tDCS and CPM protocols and found an improvement in the CPM response, but the effectiveness of the blinding was not assessed [10]. None of these studies reported the method by which M1 was localized for the tDCS [4,10].

No effect of tDCS was observed on TS as a result of tDCS. Braulio et al. (2018) reported a reduction in TS to repeated cold painful stimulus after the application of conventional M1-tDCS in subjects with remifentanil-induced hyperalgesia [10]. This could be an indication that our results are likely due to a floor effect, and that tDCS can only reverse the TS phenomenon when hyperexcitability of the spinothalamic tract is present. It is necessary to carry out more studies evaluating TS in patients with a pain pathology to better demonstrate the modulatory effects of UHCDS-tDCS. Other studies have reported a reduction of TS to repeated electric painful stimulus after conventional tDCS application, when TS is tested with a suprathreshold stimulus intensity or at high frequencies (20 Hz) [26,60]. We tested TS with repeated pressure stimulus at the pain threshold intensity, which can account for the differences reported between studies. In addition, another pilot study reported a reduction in TS to repeated heat painful stimulus [4].

There is also a controversy over the effect of tDCS on the PPT. The PPT was not modulated after UHCDS-tDCS or M1-tDCS in comparison with the sham group. Flood et al. reported an improvement [9] and no change of the PPT after HD-tDCS applied over M1 [16] in two different randomized single-blind sham trials. In a randomized double-blind sham trial, Borckardt et al. [12] found no change of the PPT after HD-tDCS application also applied over M1. Using a conventional M1-tDCS, Bachmann et al. [66] have reported an improvement of mechanical pain threshold measured using pinprick stimulators, whereas Grundmann et al. [67] found no effect of the same tDCS applied over the primary somatosensory cortex.

This study failed to find any change in cold pain intensity following either UHCDS-tDCS or M1-tDCS. In line with our findings, Flood et al. [9] reported no differences in cold hyperalgesia after HD-tDCS application, using a cold water immersion test. Neither Bachmann et al. [66] nor Grundmann et al. [67] found changes in cold pain thresholds using the thermal quantitative sensory test after M1-tDCS or S1-tDCS application. However, these studies reported that the cold detection threshold increased. The discrepancy in the results could be due to the application of different tDCS protocols or measurement tools.

Differences in isometric grip strength between sham-tDCS, M1-tDCS and UHCDS-tDCS were not detected. Previous systematic reviews have reported an increase in the maximal isometric voluntary contraction (MIVC) with a small effect size [68], and positive effects on increasing muscular strength with tDCS [69]. The studies reviewed in these systematic reviews highlight a significant methodological heterogeneity reflecting the different testing protocols used (i.e., isometric and isokinetic strength). Most of the studies applied anodal tDCS over M1, for 10 to 20 min, with an intensity of between 1.5 and 2 mA, using 12–25 cm^2^ sponge electrodes and a sham control group. Several studies did not find any improvement in MIVC when comparing M1-tDCS application with sham-tDCS [16,70,71,72]. Hazime et al. [73] found an increase in internal and external shoulder rotator MIVC in handball players. Vargas et al. [74] reported an increment in knee extension MIVC of the dominant leg but not of the non-dominant leg in soccer players. The differences in the results could be due to the recruitment of athletes in contrast to healthy adults. Another study found an improvement in MIVC of the left hallux adduction [75], but this study also showed a dropout rate of 20% of the participants, which could mean a higher risk of bias in the results. Although UHCDS-tDCS has been proven to improve cortical excitability more than M1-tDCS [76], we found no effect on maximal isometric grip strength.

UHCDS-tDCS in healthy subjects has been shown to increase corticospinal excitability by two-fold in comparison with conventional M1-tDCS [76]. Differences between UHCDS-tDCS and M1-tDCS were found for motor skill acquisition in healthy subjects [24]. Its effect has also been evaluated on motor rehabilitation in sub-acute stroke patients, with superior results using UHCDS-tDCS instead of M1-tDCS [25]. No difference was found between UHCDS-tDCS, M1-tDCS and sham-tDCS with respect to pain outcome measures in healthy subjects. This finding could be due to a ceiling effect of tDCS on CPM and TS in healthy subjects. To our knowledge, no study has demonstrated the effect of UHCDS-tDCS on musculoskeletal pain. Future studies should consider assessing the effectiveness of UHCDS-tDCS in patients with chronic musculoskeletal pain, neuropathic pain and other painful neurologic disorders. Another possible study could be to use a unihemispheric concurrent dual-site HD-tDCS to obtain more local effects on M1 and DLPFC.

- Limitations

There are several limitations to our study which require a cautious interpretation of the findings. Although, to our knowledge, our study is the first triple-blind trial that evaluates the effect of tDCS on the CPM response and TS in healthy subjects, randomization was only done for the first treatment session. After the first randomized treatment session, the sequence of the intervention protocol always followed the same order. Despite efforts to blind the subject to the intervention, a learning carryover effect between sessions could also be present. Previous studies have reported greater changes in corticospinal excitability with UHCDS-tDCS than M1-tDCS [23]. Even so, it is recommended that corticospinal excitability changes using Transcranial magnetic stimulation (TMS) are assessed to identify differences between groups and differentiate responders from non-responders. This study also assumes that there could be a ceiling effect to detecting an increase in CPM, and a flooring effect to detecting a reduction in TS in healthy subjects. As observed in the study by Braulio et al. [10], a reduction in TS was only observed in the group that received remifentanil-induced hyperalgesia with tDCS application. This finding in healthy subjects could be due to reversal of the spinothalamic tract hyperexcitability by tDCS as a result of remifentanil infusion alone. Another potential limitation of this study is the large number of pain outcome measures recorded in a short amount of time, which could generate a carryover effect from one measurement to another or a contamination effect. The most commonly used protocol for tDCS modulation of chronic pain consists of a 20 min anodal stimulation of M1 for five consecutive days [77]. In this study, the application of a single tDCS session in healthy subjects could be insufficient to induce a neuromodulatory effect of pain. A final limitation of our study is the fact that the change in cortical excitability achieved by tDCS was not corroborated. Future studies should assess change in TMS motor-evoked potentials.

## 5. Conclusions

In conclusion, this study has shown that neither UHCDS-tDCS of M1 and DLPFC nor M1-tDCS alone affected conditioned pain modulation or temporal summation in healthy subjects, when compared to sham-tDCS.

## Figures and Tables

**Table 1:** Demographic, pain and mood characteristics of recruited participants at baseline.

Characteristic	
**Age: mean (range)**	21.92 (18–29)
**Sex**	
Number of men (%)	51 (56.7)
Number of women (%)	39 (43.3)
Height (m)	1.70 ± 0.08
Weight (kg)	66.16 ± 10.27
Body mass index (kg/m^2^): mean	22.68 ± 1.90
**Smoking**	
Number of cigarettes	1.83 ± 7.72
**Pressure pain threshold (kg/cm^2^)**	
PPT_Finger	7.12 ± 2.16
PPT_Trapezius	2.53 ± 2.27
PPT_Tibial muscle	7.49 ± 2.92
Mechanical hyperalgesia (kg/cm^2^)	19.15 ± 6.46
Tolerance threshold (kg/cm^2^)	12.41 ± 1.68
Hand grip strength (kg)	35.66 ± 15.48
Cold hyperalgesia ^1^	35.15 ± 19.06
CPM (Δkg/cm^2^)	1.87 ± 1.31
Temporal summation ^1^	9.64 ± 13.59
ULNT1 (degrees)	135.57 ± 34.91
STAI-S (0–60)	10.70 ± 9.35
STAI -T (0–69)	22.97 ± 9.85
BDI-II (0–63)	4.13 ± 6.24
PCS (0–52)	6.63 ± 6.49
TSK (11–44)	17.20 ± 5.46
PSQI (0–21)	3.72 ± 2.34
FPQIII (30–150)	57.83 ± 16.23
IPAQ-SF (METS)	3835.43 ± 2332.67

^1^ Measured with a 0–100 mm pain rating visual analog scale. PPT: pain pressure threshold; CPM: conditioned pain modulation; ULNT1: upper limb neural tension test 1; STAI-S: state–trait anxiety inventory—state; STAI-T: state–trait anxiety inventory—trait; BDI-II: Beck depression index II; PCS: pain catastrophizing scale; TSK: Tampa scale for kinesiophobia; PSQI: Pittsburgh sleep quality index; FPQIII: fear of pain questionnaire III; IPAQ-SF: international physical activity questionnaire—short form, METS: metabolic equivalent of task.

**Table 2:** ANOVA comparing effects between M1-tDCS vs. UHCDS-tDCS vs. sham-tDCS on pain processing variables and strength.

	Placebo		M1-tDCS		UHCDS-tDCS		*p*-Values	
	Pre	Post	Pre	Post	Pre	Post	Time	Group
PPT								
Finger	7.04 ± 2.15	7.16 ± 2.07	7.01 ± 1.95	7.03 ± 1.68	7.32 ± 2.41	7.42 ± 2.44	0.58	0.95
Trapezius	4.65 ± 2.52	4.86 ± 2.69	4.34 ± 1.67	4.53 ± 1.78	4.61 ± 2.57	4.79 ± 2.43	<0.05 *	0.99
Tibial muscle	7.73 ± 3.02	8.30 ± 3.17	7.10 ± 2.43	8.04 ± 2.98	7.63 ± 3.30	7.96 ± 2.96	<0.05 *	0.25
Mechanical hyperalgesia	19.44 ± 6.74	20.32 ± 7.21	18.45 ± 5.26	19.61 ± 5.67	19.57 ± 7.37	20.18 ± 7.03	<0.05 *	0.76
Tolerance threshold	12.44 ± 1.59	12.67 ± 1.51	12.42 ± 1.84	12.54 ± 1.75	12.37 ± 1.65	12.21 ± 1.69	0.58	0.38
Hand grip strength	36.29 ± 15.51	36.39 ± 15.23	33.95 ± 17.79	34.85 ± 18.32	36.71 ± 13.20	37.56 ± 13.67	<0.05 *	0.44
Cold hyperalgesia	35.75 ± 21.54	31.86 ± 21.25	36.67 ± 18.13	31.06 ± 19.09	33.02 ± 17.68	32.11 ± 18.59	<0.05 *	0.17
CPM	1.62 ± 1.32	2.28 ± 1.58	2.26 ± 1.38	2.20 ± 1.71	1.75 ± 1.18	2.05 ± 1.09	0.06	0.18
Temporal Summation	8.37 ± 10.32	10.40 ± 13.28	12.00 ± 16.78	9.41 ± 13.35	9.23 ± 13.05	10.03 ± 12.92	0.95	0.35
ULNT1	137.07 ± 33.71	143.93 ± 30.75	133.63 ± 34.31	143.97 ± 34.1	136.00 ± 37.68	141.24 ± 32.94	<0.05 *	0.54

PPT: pain pressure threshold; CPM: conditioned pain modulation; ULNT1: upper limb neural tension test 1 (* *p* < 0.05).

## Data Availability

No new data were created or analyzed in this study. Data sharing is not applicable to this article.
